# MICAL-mediated oxidation of actin and its effects on cytoskeletal and cellular dynamics

**DOI:** 10.3389/fcell.2023.1124202

**Published:** 2023-02-17

**Authors:** Sudeepa Rajan, Jonathan R. Terman, Emil Reisler

**Affiliations:** ^1^ Department of Chemistry and Biochemistry, University of California, Los Angeles, Los Angeles, CA, United States; ^2^ Departments of Neuroscience and Pharmacology, University of Texas Southwestern Medical Center, Dallas, TX, United States; ^3^ Molecular Biology Institute, University of California, Los Angeles, Los Angeles, CA, United States

**Keywords:** MICAL1, MICAL2, MICAL3, MsrB, SelR, semaphorin, plexin, rab

## Abstract

Actin and its dynamic structural remodelings are involved in multiple cellular functions, including maintaining cell shape and integrity, cytokinesis, motility, navigation, and muscle contraction. Many actin-binding proteins regulate the cytoskeleton to facilitate these functions. Recently, actin’s post-translational modifications (PTMs) and their importance to actin functions have gained increasing recognition. The MICAL family of proteins has emerged as important actin regulatory oxidation-reduction (Redox) enzymes, influencing actin’s properties both *in vitro* and *in vivo*. MICALs specifically bind to actin filaments and selectively oxidize actin’s methionine residues 44 and 47, which perturbs filaments’ structure and leads to their disassembly. This review provides an overview of the MICALs and the impact of MICAL-mediated oxidation on actin’s properties, including its assembly and disassembly, effects on other actin-binding proteins, and on cells and tissue systems.

## 1 Introduction

Actin is one of the main components of the cytoskeleton, playing an essential role in muscle contraction, cell division, motility, navigation, mechanosensing, and in maintaining cellular structure (Blanchoin et al., 2014; Pollard, 2016; Rottner et al., 2017; Lappalainen et al., 2022). Actins share high sequence homology (∼90%) across species from invertebrates to vertebrates. Six distinct isoforms of actin are present in invertebrates such as *Drosophila* and vertebrates (called α-cardiac, α-skeletal, α-smooth, γ-smooth, β-cytoplasmic, and γ-cytoplasmic actins in mammals), which vary primarily at their N-terminus (Kashina, 2020). The actin cytoskeleton is dynamic and constantly remodels to perform various cellular functions (Blanchoin et al., 2014; Pollard, 2016; Lappalainen et al., 2022). Many actin-binding proteins (ABPs) dynamically fine-tune actin structures in response to cell needs (Gupta et al., 2020; Kadzik et al., 2020; Lappalainen et al., 2022). These proteins help in actin assembly from its monomers (G-actin) to filaments (F-actin) and then to higher-order structures, such as actin bundles and networks (Pollard, 2016; Merino et al., 2020; Gautreau et al., 2022). Among others, ABPs are categorized as proteins assisting in actin nucleation and elongation (e.g., formins and Ena/VASP family), network formation (e.g., Arp2/3 complex), severing and depolymerization (e.g., ADF/cofilin, gelsolin superfamily, and twinfilin), bundling (e.g., fascin, espin, α-actinin), G-actin binding (e.g., profilin), and barbed-end/pointed-end capping (e.g., CapZ and tropomodulin) (Siripala and Welch, 2007a; Siripala and Welch, 2007b; Pollard, 2016; Lappalainen et al., 2022).

It is now well-established that besides classical ABPs that regulate actin’s properties and dynamics *via* physical, non-covalent mechanisms, actin is also regulated by alterations of its amino acids (Terman and Kashina, 2013; Varland et al., 2019). These co-translational and post-translational modifications (PTMs) of actin, such as phosphorylation, oxidation, acetylation, arginylation, SUMOlytion, ubiquitination, and others, also dynamically control actin’s properties, including F-actin’s stability, functions, and interaction with other ABPs (Terman and Kashina, 2013; Varland et al., 2019). Since actin is one of the most ubiquitous proteins, some of these PTMs are likely to occur non-specifically, including as by-products of enzymatic reactions or *via* non-enzymatic (including environmental) mechanisms (Terman and Kashina, 2013; Varland et al., 2019). Yet, specific enzymes have also now been identified that selectively target actin as a substrate. Defining these enzymes and their modification of actin is therefore a critical biomedical goal.

MICAL (Molecule Interacting with CasL) proteins have recently emerged as a family of enzymes that selectively target actin as a substrate. MICALs specifically bind and oxidize F-actin to posttranslationally alter specific amino acids within filament subunits, and by this, MICALs robustly alter the properties of actin (Hung et al., 2010a; Hung et al., 2011). Herein, we review the MICALs, first describing their protein organization, enzymatic region, and structural properties. Then, we highlight MICAL-mediated post-translational oxidation of actin and its impact on actin structures and dynamics. Lastly, we focus on the modulation of MICAL-mediated actin regulation by other proteins and the consequence of the MICAL’s oxidation of actin on cellular and tissue functions and dysfunctions.

## 2 The MICAL family of proteins

**FIGURE 1:**
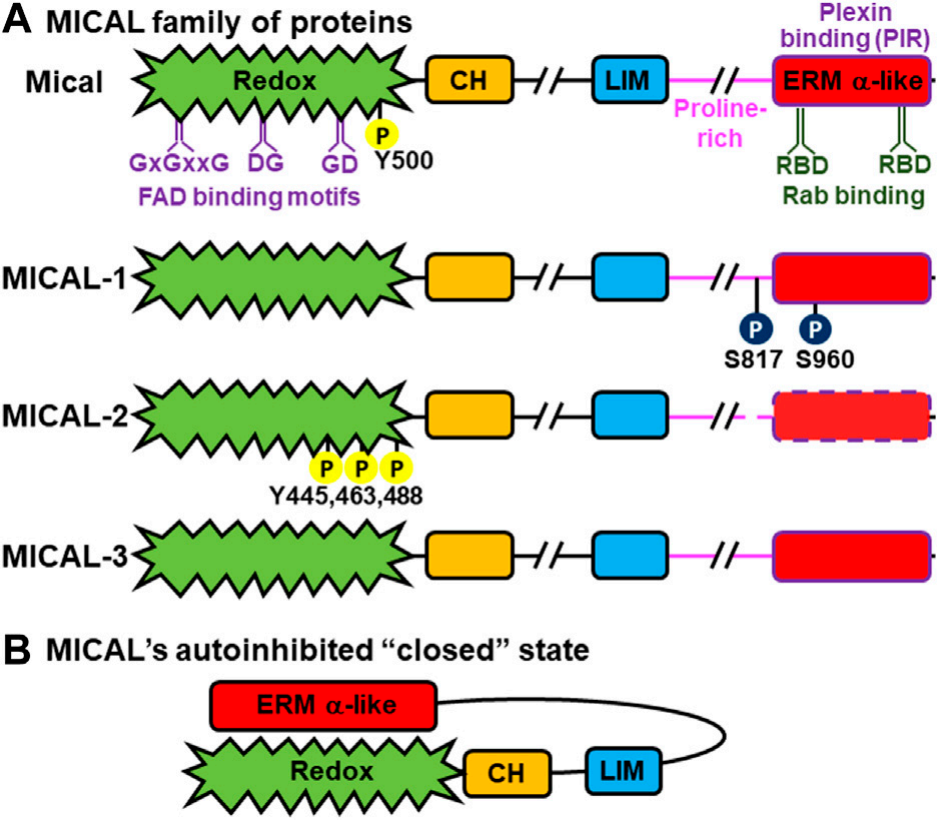
MICAL family proteins: domain organization and allosteric interaction. **(A)** Domain organization of *Drosophila* Mical and human/mammalian MICAL-1, MICAL-2, and MICAL-3. All MICALs contain the N-terminal flavoprotein monooxygenase (Redox) domain (green), followed by a calponin homology (CH) domain (orange), a LIM domain (blue), a proline-rich region (pink), and an ERM alpha (α)-like domain (red). These domains are linked by regions of variable length (//). The FAD binding (GxxGxxG, DG, and GD) motifs in the Redox domain are denoted in purple. The two Rab binding (RBD) regions (dark green) and plexin binding (PIR) region (purple) within the ERM α-like domain are also shown. Sites of phosphorylation by Abl kinase are shown as yellow circles [Y (tyrosine)], while those of PAK1 kinase are in dark blue circles [S (Serine)]. It is notable that many annotated cDNAs for MICAL-2 do not contain the C-terminal ERM α-like region, but the *MICAL-2* genomic locus includes an ERM α-like region that is similar to MICAL-1 and MICAL-3. This region has independently been called MICAL-CL and Ebitein and is denoted here with dashed lines. **(B)** Full-length MICAL family members have been found to exist in autoinhibited (inactive) forms. In the autoinhibited form, the C-terminus ERM α-like domain of the MICAL’s folds in antiparallel fashion towards the N-terminus, and interacts with the Redox and LIM domains to inhibit the Redox enzymatic activity of the MICALs.

MICAL proteins were found independently as a binding partner for the SH3-domain containing adaptor protein CasL (Suzuki et al., 2002) and as a functional driver of cellular morphology/neuronal guidance downstream of Plexin cell-surface receptors (Terman et al., 2002). It was the work in (Terman et al., 2002) that first identified MICALs as having an enzymatic domain and indicated they were oxidation-reduction (Redox) enzymes. That work (Terman et al., 2002) also identified multiple different *MICAL* genes and that they comprise a family of phylogenetically-conserved proteins with one member in *Drosophila* (called Mical) and three in vertebrates, including humans [named MICAL-1, MICAL-2, and MICAL-3 (or also known as MICAL1, MICAL2, and MICAL3)] (Figure 1A). MICALs are large proteins (>1,000 amino acids) consisting of a highly conserved N-terminal flavoprotein monooxygenase (also called hydroxylase, MO, FM, or Redox) domain, followed by several other notable regions, including a calponin homology (CH) domain, a Lin-11, Isl-1, Mec-3 (LIM) domain, a proline-rich region with PxxP ligands for SH3-domain containing proteins, and a region that shares homology to the alpha (*α*) region of Ezrin, Radixin, and Moesin (ERM) proteins (this region is now often referred to as the Plexin-interacting region (PIR), Rab binding domain (RBD), bMERB, or CC) [Figure 1A (Terman et al., 2002; Alto and Terman, 2018; Rouyère et al., 2022)]. Each MICAL family member, including those in *Drosophila* and humans, has multiple different splice forms/isoforms (Terman et al., 2002), which impact their enzymatic and cellular functions [Figure 1A; e.g., (Terman et al., 2002; Weide et al., 2003; Pasterkamp et al., 2006; Hung and Terman, 2011; Wilson et al., 2016; Rouyère et al., 2022)]. Among the different family members, the MICALs are expressed in most, if not all, tissues (see below).

The Redox region of the MICALs consists of three motifs that bind specifically to FAD (flavin adenine dinucleotide): a distinct dinucleotide binding Rossman fold GxGxxG motif (where G is glycine and x is any amino acid), and both GD (glycine-aspartic acid) and DG (aspartic acid-glycine) motifs that interact with the ribose and pyrophosphate moieties of flavin, respectively [Figure 1A (Terman et al., 2002)]. The presence of these three motifs defined MICALs as flavoprotein monooxygenases, *versus* other types of Redox enzymes such as oxidases (Terman et al., 2002). Structural studies have also confirmed that the Redox region of MICALs is most similar to flavoprotein monooxygenases, including to the classical flavoprotein monooxygenase *p*-hydroxybenzoate hydroxylase (*p*HBH) (Nadella et al., 2005; Siebold et al., 2005; Alqassim et al., 2016; Kim et al., 2020). Biochemical assays have also confirmed MICALs’ catalytic activity. In particular, MICALs non-covalently bind the cofactor FAD and use NADPH as a coenzyme and oxygen (O_2_) in Redox reactions (Terman et al., 2002; Nadella et al., 2005; Hung et al., 2010a; Hung et al., 2011; Zucchini et al., 2011; McDonald et al., 2013; Wu et al., 2018). Further, unlike oxidases, flavoprotein monooxygenases such as *p*HBH have substrates that they physically interact with, are activated by, and specifically modify [i.e., direct substrates (Huijbers et al., 2014)]. So too, MICALs’ Redox region has substrates that they physically interact with and whose binding activates MICALs to specifically modify them. In particular, MICALs modify specific methionine residues within these direct substrates: F-actin being the best defined [(Hung et al., 2011); see below]. Of note, in this way, MICAL family proteins are the first flavoprotein monooxygenases found to have a direct protein substrate. Another protein, the Ca2+/calmodulin-dependent protein kinase II (CaMKII), has also recently been linked to being a direct substrate for MICAL-1 (Konstantinidis et al., 2020). In the absence of a substrate, flavoprotein monooxygenases such as *p*HBH produce hydrogen peroxide (H_2_O_2_) (Entsch and van Berkel, 1995; Huijbers et al., 2014), and can therefore modify molecules indirectly (i.e., as indirect substrates). The MICALs’ Redox domain also produces H_2_O_2_ in the absence of a direct substrate (Terman et al., 2002; Nadella et al., 2005; Schmidt et al., 2008; Hung et al., 2010a; Hung et al., 2011; Zucchini et al., 2011; McDonald et al., 2013; Wu et al., 2018). This H_2_O_2_ affects the activity of the collapsin response mediator protein (CRMP) (Schmidt et al., 2008; Morinaka et al., 2011; Tominaga et al., 2019) and the tau protein (Prifti et al., 2022), with which MICALs are known to interact, by oxidizing specific cysteine residues in them (Schmidt et al., 2008; Morinaka et al., 2011; Tominaga et al., 2019; Prifti et al., 2022). A member of the actin nucleator Arp2/3 complex, Arp3B, has also been linked to being modified by the MICALs on a specific methionine residue (Galloni et al., 2021), but it has not yet been determined whether this is *via* direct or indirect mechanisms. It is also notable that the amount of H_2_O_2_ produced differs between different MICALs, such that MICAL-1 produces higher amounts of H_2_O_2_, as compared to other known monooxygenases and other MICALs (Nadella et al., 2005; Zucchini et al., 2011; Wu et al., 2018). In this regard, MICAL-1 has a substitution of a critical aspartate (Asp) to alanine (Ala) in the flavin-binding DG motif, which is responsible for this increased catalytic activity in the absence of a substrate (Wu et al., 2018).

Unlike classical flavoprotein monooxygenases such as *p*HBH, MICALs contain other domains besides their Redox domains. Therefore, MICALs are referred to as multidomain flavoprotein monooxygenases (Terman et al., 2002) and these other domains, including the proteins they interact with, are essential for regulating the activity of the MICAL’s Redox domain (Schmidt et al., 2008; Hung et al., 2010a; Alto and Terman, 2018; Rouyère et al., 2022). In particular, the MICAL’s other domains have been found to regulate its catalytic activity and its effects in cells*/in vivo* – including to induce it to exist in an autoinhibited state, such that the C-terminal portion of the MICALs autoinhibits their N-terminal Redox activity [Figure 1B; (Schmidt et al., 2008; Hung et al., 2010a; Giridharan et al., 2012; Vitali et al., 2016; Frémont et al., 2017)]. The MICAL’s Redox activity therefore is under the spatiotemporal instruction of other specific proteins (Terman et al., 2002; Schmidt et al., 2008; Hung et al., 2010a; Frémont et al., 2017) (see below).

The CH domain of each of the MICALs (Figure 1A) is structurally similar to the CH type-2 domain present in several other actin-binding proteins (such as smoothelin) (Terman et al., 2002). While CH type-1 domains are known to directly bind to F-actin, CH type-2 domains, including those present in MICALs, do not appear to directly bind to F-actin (Sun et al., 2006; Ishida et al., 2008; Yin et al., 2020). Indeed, work with purified proteins has revealed that the Redox domain of the MICALs alone is sufficient for its binding to F-actin (Hung et al., 2010a; Yoon et al., 2021). So too, the catalytic activity (Kcat) of the MICAL’s Redox domain and its effects on F-actin are similar with or without the CH domain (Hung et al., 2010a; Vitali et al., 2016; Yoon et al., 2021). Yet, the MICAL’s CH domain may help facilitate the MICAL’s F-actin binding, including the possibility that the MICAL’s CH domain might interact with the CH domain of other proteins to further promote the MICAL’s F-actin binding (Alqassim et al., 2016; Kim et al., 2020). The MICAL’s CH domain may also provide the means to interact with non-CH domain-containing proteins (Suzuki et al., 2002). Based on analogy to other proteins, the MICAL’s CH domain (or residues nearby) may also be involved in its self-association (e.g., (Liu et al., 2016; Rai et al., 2020)). Notably, *in vivo* work reveals that the CH domain helps localize the MICALs subcellularly (through unknown means) to exert its F-actin effects (Hung et al., 2010a).

The LIM domain of each of the MICALs (Figure 1A) is highly similar to other LIM domains, indicating it contains Zn2+ finger motifs (Terman et al., 2002). As with other LIM domains, the MICAL’s LIM domain may serve to bring it into contact with other proteins (Matthews et al., 2009) — including that it may contribute to the MICAL’s interaction with CRMP, although no direct interactions between MICALs’ LIM domain and CRMP have been observed (Schmidt et al., 2008). The MICAL’s LIM domain may also be involved in its self-association (Schmidt et al., 2008; Liu et al., 2016; Miyake et al., 2019). The MICAL’s proline-rich PxxP region (Figure 1A) is known to mediate its interactions with other proteins, including with SH3 domain-containing proteins such as the adaptor protein CasL (Suzuki et al., 2002) and the non-receptor tyrosine kinase Abl (Yoon et al., 2017). The MICAL’s C-terminal ERM alpha (α)-like domain (also called PIR, CC, RBD, bMERB) [Figure 1A (Terman et al., 2002; Alto and Terman, 2018; Rouyère et al., 2022)] serves an essential function in regulating the enzymatic activity of the MICALs. It is through this region that MICALs interact with the cytoplasmic portion of the Plexin transmembrane receptor (Terman et al., 2002; Schmidt et al., 2008) and also with the activated (GTP-bound form) of Rab GTPases (Weide et al., 2003; Fukuda et al., 2008; Deng et al., 2016; Rai et al., 2016; Frémont et al., 2017). It is also notable that many annotated cDNAs for MICAL-2 do not contain the C-terminal ERM α-like region, but the *MICAL-2* genomic locus includes an ERM α-like domain that is similar to invertebrate Mical, MICAL-1, and MICAL-3 (Terman et al., 2002; Hung et al., 2011; Giridharan and Caplan, 2014; Alto and Terman, 2018; Rouyère et al., 2022). This region has independently been called MICAL-CL and Ebitein (Figure 1A, dashed box; see (Hung and Terman, 2011) for further discussion).

Another family of proteins known as the Mical-like (MICAL-L) has also been identified (Terman et al., 2002). They share similar domain composition with the MICALs but lack the Redox domain. Humans have two MICAL-like proteins [MICAL-L1 and MICAL-L2 (also called JRAB)], while *Drosophila* has one Mical-like protein (Terman et al., 2002). MICAL-Ls have key cellular functions, including in endocytosis and vesicle trafficking [e.g., (Farmer et al., 2021; Malinova et al., 2021; Sikora et al., 2021)], but since they do not have the actin-modifying Redox domain, they will not be further discussed herein.

## 3 The MICAL’s effects on F-actin properties

**FIGURE 2:**
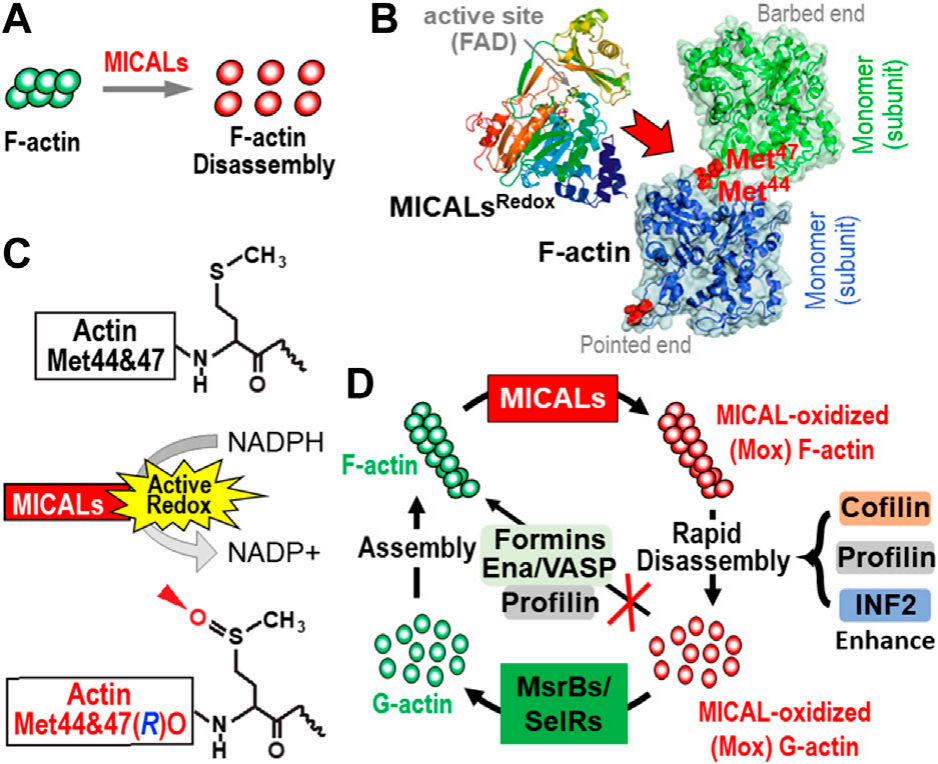
The MICAL’s activity and its effects on actin dynamics. **(A–C)** MICALs posttranslationally modify specific methionine (Met) residues in F-actin (red), which triggers F-actin disassembly **(A)**. More specifically, MICALs bind to F-actin [**(B)**, red arrow)] and in the presence of their coenzyme, NADPH **(C)**, selectively and stereospecifically oxidize (O) actin’s Met44 and Met47 in the *R*-conformation [**(C)**, arrowhead]. This oxidation of Met44 and Met47 [see **(B)**, red] occurs in the D-loop, at the pointed end of individual actin filament subunits, which disrupts the interprotomers interactions in F-actin and leads to their rapid disassembly. **(D)** MICAL-mediated F-actin disassembly is regulated by other proteins. From top and clockwise: following MICALs’ oxidation of F-actin to generate MICAL-oxidized (Mox) F-actin, MICAL-triggered F-actin disassembly is enhanced by other proteins, including cofilin, profilin, and INF2. The Mox-G-actin that is formed does not readily re-polymerize even in the presence of profilin, formins, and Ena/VASP. Yet, Mox-G-actin is reduced specifically by selective methionine sulfoxide reductases (MsrBs/SelRs), and this G-actin can then re-polymerize normally. In this way, MICALs and MsrBs/SelRs create a reversible system for Redox regulation of actin dynamics in cells.

The Redox region of all MICALs directly interacts with F-actin (Hung et al., 2010a; Hung et al., 2011; Zucchini et al., 2011; McDonald et al., 2013; Wu et al., 2018) (Figures 2A, B). Moreover, this binding to F-actin substantially enhances each MICAL family member’s ability to interact with and consume its coenzyme NADPH (Hung et al., 2011; Zucchini et al., 2011; McDonald et al., 2013; Wu et al., 2018) (Figure 2C). In particular, the direct binding of each MICAL family member to F-actin (not to G-actin) substantially increases its Redox activity (e.g., including >100-fold, in an F-actin and MICALs concentration-dependent manner) (Hung et al., 2011; Zucchini et al., 2011; McDonald et al., 2013; Wu et al., 2018). Each MICAL family member then oxidizes two specific methionine (Met) residues in actin (Met44 and Met47) in a stereoselective manner (in the Met *R*-isomer conformation) to generate actin Met-44, 47-*R*-sulfoxide [actin Met(R)O-44,47] [Figures 2B, C (Hung et al., 2011; Hung et al., 2013; Lee et al., 2013; Wu et al., 2018)]. Notably, it is the stereospecific oxidation of these two Met residues that underlies the MICALs’ F-actin effects. In particular, a high rate of actin disassembly is achieved at very low, substoichiometric levels of MICALs, pointing to the catalytic activity of MICALs in disassembling F-actin (Hung et al., 2011; Wu et al., 2018). So too, work with purified proteins and *in vivo* reveals that mutating the Met44 and Met47 residues of actin prevents MICALs from exerting their effects on F-actin (Hung et al., 2011; Lundquist et al., 2014; Grintsevich et al., 2017; Orr et al., 2017; Wu et al., 2018).

The identification of the MICALs and their mechanism for affecting actin also provides new insight into the regulation of actin by oxidative means. In particular, oxidation has long been known to affect actin filament dynamics [Reviewed in (Terman and Kashina, 2013)]. What was not clear was whether these effects were specific, selective, and/or locally controlled *versus* simply being a random by-product of enzymatic reactions or non-enzymatic mechanisms (Shacter, 2000; Stadtman et al., 2003; Kim and Gladyshev, 2007). The identification, as described above, that the MICAL’s enzymatic modification of actin is substrate specific (i.e., F-actin, but not G-actin), residues specific (i.e., Met44, Met47), and stereo specific (i.e., in the *R*-isomer *versus* the *S*-isomer conformation), thus reveals that actin dynamics are controlled by distinct oxidative mechanisms. In the same way, the MICALs do not simply release a diffusible oxidant, such as H_2_O_2_, to cause widespread effects on F-actin – since such oxidants, including H_2_O_2_, do not mimic the effects of the MICALs [e.g., cell-lethal levels of H_2_O_2_ have no effect on F-actin disassembly in the assays used to define the MICALs (Hung et al., 2010a; Hung et al., 2011; Wu et al., 2018)]. Furthermore, the H_2_O_2_ scavenger catalase (and other types of reductants such as DTT and thioredoxin/thioredoxin reductase) do not alter the MICAL’s F-actin effects (Hung et al., 2011; Hung et al., 2013). Additionally, the MICAL’s substrate residues (Met44 and Met47) are buried within F-actin, in a hydrophobic pocket that is poorly accessible to diffusible oxidants (Dalle-Donne et al., 2002; Guan et al., 2003; Guan et al., 2005; Takamoto et al., 2007; Hung et al., 2011; Chou and Pollard, 2019). Moreover, MICALs need to be in close proximity to F-actin to exert their effects (Hung et al., 2011) — and such binding to F-actin is needed to activate MICALs’ enzymatic activity (Hung et al., 2010a; Hung et al., 2011; Zucchini et al., 2011; McDonald et al., 2013; Wu et al., 2018) to then oxidize actin filament subunits (Hung et al., 2011; Wu et al., 2018). Thus, the MICAL family forms a class of monooxygenases that modify actin by direct interactions. Moreover, as the MICALs directly/physically interact with F-actin, further work will seek to capture/determine whether the MICALs directly interact with the Met44 and Met47 residues to add oxygen to them [which would be similar to how flavoprotein monooxygenases such as *p*HBH work on their substrate residues (Entsch and van Berkel, 1995; Huijbers et al., 2014)]. MICALs’ ability to stereospecifically modify Met44 and Met47 in the *R*, but not the *S*, conformation indicates this type of selective modification within the MICALs’ – F-actin substrate interaction pocket. Alternatively, the substrate binding pocket between the MICALs and F-actin (i.e., the *in situ* enzyme–substrate interaction region) may provide an environment where MICALs release an oxidant that selectively modifies Met44 and Met47.

The Met44 and Met47 residues are present in the DNaseI-binding loop (D-loop) of actin at the pointed-end of actin filament subunits (Figure 2B). Actin’s D-loop is essential for forming longitudinal contacts between actin subunits in F-actin and regulates filaments’ stability (Oztug Durer et al., 2010; Dominguez and Holmes, 2011; Durer et al., 2012; Chou and Pollard, 2019; Das et al., 2020). More specifically, the side chain of Met44 is thought to be an important residue for inter-subunit interactions along the long-pitch helix of filaments (Oztug Durer et al., 2010; Dominguez and Holmes, 2011; Durer et al., 2012; Chou and Pollard, 2019; Chou and Pollard, 2020; Das et al., 2020). Oxidation of these two methionine residues increases the local charge of F-actin (Hung et al., 2011) and alters the filaments’ D-loops positions (Grintsevich et al., 2017). This loosening of inter-subunit interactions upon MICAL-mediated oxidation destabilizes the F-actin structures, making them prone to fragmentation even under a mild mechanical force such as pipetting, and ultimately disassembles them (Hung et al., 2011; Grintsevich et al., 2016; Grintsevich et al., 2017). Of note, actin’s Met44 and Met47 are phylogenetically conserved in all actin isoforms, suggesting that MICALs are likely to exert similar effects on all actins (Hung et al., 2011). Furthermore, the importance of Met44 in actin structures can be judged by the fact that a mutation of Met44 is lethal in yeast (Oztug Durer et al., 2010). Likewise, dominant *de novo* (heterozygous missense) mutations in actin’s Met44 and Met47 result in human diseases, including those defined by an accumulation of F-actin (Laing et al., 2009; Hung et al., 2010b; Hoffjan et al., 2011; Zou et al., 2013; Regalado et al., 2014; Wangler et al., 2014; Yates et al., 2017; Zhang et al., 2019).

The MICAL-mediated modification of F-actin subunits induces filaments to disassemble [Figure 2A (Hung et al., 2010a; Hung et al., 2011; Wu et al., 2018)]. Real-time analysis of immobilized actin filaments by TIRF microscopy reveals that MICALs induce depolymerization and severing (fragmentation) of filaments [e.g., (Hung et al., 2011; Grintsevich et al., 2016; Grintsevich et al., 2017)]. MICALs depolymerize barbed ends of filaments with disassembly rates that reach as high as >84 subunits/sec, as opposed to disassembly rates that on rare occasions can reach as high as ∼5.4 subunits/sec for unmodified F-actin (Grintsevich et al., 2017). At the same time, the pointed-end depolymerization rate is ∼1.44 subunits/sec in the presence of MICAL, in contrast to ∼0.17 subunits/sec in control (no MICAL) conditions (Frémont et al., 2017; Grintsevich et al., 2017). Thus, the prime cause of MICAL-oxidized F-actin’s rapid depolymerization are their unstable barbed ends. Intriguingly, the nucleotide state of F-actin also plays a considerable role in its susceptibility to MICAL-mediated depolymerization. ADP-Pi·F-actin is less prone to MICAL-mediated depolymerization than ADP·F-actin (Grintsevich et al., 2017). Incubation of MICAL-oxidized F-actin with either BeFx (which mimics the ADP-Pi cap at the barbed ends) or a heterodimeric capping protein (CP) abolishes the rapid depolymerization event (Grintsevich et al., 2017). Overall, this reveals that MICALs affect aged actin filaments much more than new ones. This is significant at the cellular level because rapid actin remodeling is required to bring morphological changes to cells – and these changes are achieved by disassembling existing actin structures (filaments and bundles). Indeed, MICALs dramatically decrease and remodel F-actin structures in cultured cells and *in vivo*, both in the cytosol [(Hung et al., 2010a; Hung et al., 2011; Hung et al., 2013; Lee et al., 2013; Wu et al., 2018); see below] and in the nucleus (Lundquist et al., 2014) — and exert disassembling effects on different networks of actin including bundled actin (Hung et al., 2010a), branched actin (Galloni et al., 2021), and actin bound to decorating proteins — including tropomyosin (Wioland et al., 2021). Loss of MICALs *in vivo* results in the accumulation of F-actin [e.g., (Beuchle et al., 2007; Hung et al., 2010a; Hung et al., 2011; Hung et al., 2013; Lundquist et al., 2014); see below].

Actin disassembly proteins, such as cofilin, gelsolin, twinfilin, *etc.*, sever or depolymerize filaments by inducing conformational changes in F-actin upon their binding (Pollard, 2016; Lappalainen et al., 2022). MICALs, on the other hand, chemically modify (oxidize) actin filaments to cause their severing and depolymerization (Hung et al., 2010a; Hung et al., 2011; Grintsevich et al., 2016; Frémont et al., 2017; Grintsevich et al., 2017; Wu et al., 2018). Additionally, in cofilin-mediated severing/depolymerization, the end products are newly formed barbed ends or G-actin, which promote actin polymerization (Chin et al., 2016). In contrast to that, the end product of the MICAL’s disassembly is MICAL-oxidized actin – and it is ineffectively reused for filaments’ elongation due to its poor polymerization properties (Hung et al., 2011; Hung et al., 2013; Lee et al., 2013; Wu et al., 2018; Grintsevich et al., 2021). Thus, in contrast to other well-known actin-binding proteins that disassemble F-actin but also promote actin polymerization, MICALs both disassemble F-actin and inhibit actin polymerization.

## 4 Structural and dynamic properties of MICAL-oxidized actin (Mox-actin)

MICAL-oxidized actin (Mox-actin) is the end product of the site-specific F-actin oxidation by the MICALs. Mox-actin has different polymerization properties than unoxidized actin. ATP-bound Mox-actin monomers (Mox-G-actin) form filaments with altered polymerization kinetics and length compared to unoxidized actin. They have ∼10-fold higher critical concentration for polymerization than unoxidized actin (∼1 μM for Mox-actin *versus* 0.1 μM for unoxidized actin), with a prolonged nucleation phase that reaches saturation at a lower level than unoxidized actin (Grintsevich et al., 2016). This results in ∼3-fold slower rate of Mox-G-actin elongation than that of unoxidized actin. Intriguingly, ADP-bound Mox-actin monomers cannot polymerize even at high concentrations (>30 µM) (Grintsevich et al., 2017). Notably, a decrease in the critical concentration of Mox-actin was observed when BeFx was present (∼0.24 µM), suggesting that the protection of barbed ends of filaments reduces their disassembly (Grintsevich et al., 2017). In addition to that, the filaments formed by Mox-actin are also generally very short and highly fragile, breaking even under small force as mentioned above (Grintsevich et al., 2016; Grintsevich et al., 2021). Altogether, Mox-G-actin is unlikely to participate in active actin polymerization in the cell, and even if it does, those filaments would not be stable enough to withstand any applied force.

Mox-F-actin also readily disassembles — by steps of slow (∼2.6 subunits/sec compared to ∼0.2 subunits/sec for unmodified actin) and catastrophic depolymerization (>84 subunits/sec compared to rare events of ∼5.4 subunits/sec for unmodified actin) (Grintsevich et al., 2017). Cryo-EM structural analysis showed two Mox-F-actin conformations (Class 1, PDB ID: 6AV9, and Class 2 PDB ID: 6AVB), supporting the two different disassembly modes (Grintsevich et al., 2017). Most of the structural changes were limited to the D-loop. In Class 1, Met44-O (MICAL-oxidized Met44) moves out from its canonical position (hydrophobic cleft of actin), which ablates the interprotomer longitudinal contacts in Mox-F-actin. In contrast to that, Met47-O in Class 1 forms a new hydrogen bond with threonine (Thr) 351, thereby twisting the D-loop and further destabilizing F-actin (Grintsevich et al., 2017). Thus, the Class 1 conformation supports the catastrophic (>84 subunits/sec) disassembly of F-actin (Grintsevich et al., 2017). In Class 2, Met44-O remains in its position and forms close interactions with the cleft. Met47-O in this class forms a hydrogen bond with tyrosine (Tyr) 169 instead, and the Mox-F-actin structure remains similar to that of unmodified F-actin (Grintsevich et al., 2017). These structural observations complement the enzymology and site-directed mutagenesis results described above, i.e. that Met44 is the main site through which MICAL-mediated oxidation exerts its effects on F-actin but Met47 oxidation also impacts F-actin (Hung et al., 2011; Lundquist et al., 2014; Grintsevich et al., 2017; Orr et al., 2017; Wu et al., 2018) and support the notion that MICALs rapidly disassemble F-actin by modifying the Met44 and Met47 residues present in the D-loop of F-actin subunits.

## 5 The MICAL’s and Mox-actin’s effects on the function of actin-binding proteins, and *vice versa*

The MICAL’s Redox actin regulatory system is therefore an unusual system for robustly regulating actin dynamics. Yet, recent results also reveal that it should not be thought of as working independently from other classical actin-binding proteins but that it interacts/works together with them. In particular, the binding of ABPs to F-actin is controlled by its nucleotide-bound state (Chou and Pollard, 2019), its tension and torque (Hayakawa et al., 2011; Risca et al., 2012), and structural impacts due to the presence of other actin-binding proteins (Reymann et al., 2012; Ngo et al., 2016; Mizuno et al., 2018). Furthermore, since MICALs oxidize F-actin specifically at its two Met residues in the D-loop (Hung et al., 2011), this alters F-actin’s structure (Grintsevich et al., 2017) and, thereby F-actin’s interactions with other ABPs (see below).

Cofilin is a well-studied actin depolymerization and severing protein. It binds cooperatively to actin filaments to form clusters of cofilin-decorated regions (Wioland et al., 2017). The boundaries between these clusters are the filaments’ severing sites (Andrianantoandro and Pollard, 2006; Suarez et al., 2011). Severing occurs because the cofilin-decorated regions are over-twisted compared to the bare F-actin, leading to its structural instability (Bobkov et al., 2004; Bobkov et al., 2006; Huehn et al., 2020). MICAL-mediated oxidation of actin subunits weakens inter-longitudinal interactions in F-actin and makes them more fragile and susceptible to severing/depolymerization-inducing conditions [Figure 2D (Hung et al., 2011; Grintsevich et al., 2016; Grintsevich et al., 2017)]. Even low concentrations of cofilin, which are harmless to unmodified F-actin, disrupt Mox-F-actin by quickly disassembling it [Figure 2D (Grintsevich et al., 2016; Wioland et al., 2021)]. Thus, despite cofilin having a low affinity for ADP. Pi-actin, and/or the presence of inorganic phosphate inhibiting F-actin depolymerization, the MICAL’s oxidation of F-actin counteracts these effects (Muhlrad et al., 2006; Grintsevich et al., 2017; Wioland et al., 2021). MICALs do this by increasing cofilin’s binding and favoring the growth of cofilin domains on actin filaments (Grintsevich et al., 2016; Wioland et al., 2021). The MICAL’s oxidation of actin allows cofilin to exert its effects also in the presence of at least some of its well-known inhibitory modifications, such as a phosphomimetic substitution at Ser3 [that is thought to mimic the effects of phosphorylation by LIM Kinase, a well-known inhibitor of cofilin (Wioland et al., 2021)].

Profilin, a well-known G-actin binding protein, also increases F-actin depolymerization (Kinosian et al., 2002; Jégou et al., 2011; Courtemanche and Pollard, 2013). Interestingly, its depolymerization effect is more profound on Mox-F-actin, for which even low profilin concentrations are sufficient to induce its disassembly [Figure 2D (Grintsevich et al., 2021)]. The exact mechanism for how profilin causes this increased disassembly is not known, but several mechanisms are in line with the data (Grintsevich et al., 2021). In particular, modeling studies suggest that profilin’s binding to the barbed-end of F-actin requires reduced flattening of terminal actin subunits to prevent steric clashes (Courtemanche and Pollard, 2013). This effect would increase the rate of subunits dissociation from the barbed ends of actin by inducing more of a G-actin-like conformation – and thus further enhance the depolymerization of Mox-actin, which is known to be substantially faster than “normal” actin even in the absence of profilin (Grintsevich et al., 2017; Grintsevich et al., 2021). It is also possible, given the unusual structure of Mox-F-actin (Grintsevich et al., 2017), that free profilin binds to Mox-actin’s barbed-ends with higher affinity compared to “normal” actin, therefore, blocking new subunit addition (Kinosian et al., 2002; Jégou et al., 2011; Courtemanche and Pollard, 2013). Furthermore, a loss of an ATP cap at the barbed-end of Mox-F-actin may also contribute to profilin-induced destabilization of Mox-F-actin by exposing ADP-bound Mox-F-actin segments that are intrinsically unstable and undergo catastrophic collapse (Grintsevich et al., 2017). This intrinsic/profilin-induced instability of Mox-F-actin at its barbed ends would also be enhanced if the association of profilin–Mox-ATP-G-actin complexes with barbed ends is greatly inhibited/abolished.

The inverted formin (INF2) is an atypical formin that can both polymerize and depolymerize actin in a concentration-dependent manner (Chhabra and Higgs, 2006; Gurel et al., 2014; Hegsted et al., 2017). Mox-F-actin is also more vulnerable to INF2 [the non-autoinhibited form of INF2 (INF2-FFC)]-mediated depolymerization than unoxidized actin [Figure 2D (Das et al., 2022)]. The exact mechanism of Mox-F-actin disassembly by INF2-FFC is unknown, but it could be due to an INF2-induced enhanced destabilization of its D-loop upon MICAL-mediated oxidation, and a further weakening of interprotomer actin contacts in Mox-F-actin (Grintsevich et al., 2017). Overall, the oxidation of filaments by the MICALs sensitizes them for rapid disassembly by other more classical severing/depolymerizing actin-binding proteins (Figure 2D).

Mox-G-actin is the end product of MICAL-mediated rapid F-actin disassembly — with or without the assistance of other actin severing/depolymerizing proteins. In cells, G-actin is the fuel for actin polymerization and is required for maintaining cellular structures. Formins and Ena/VASP are actin nucleation/polymerization-promoting proteins that bind directly, or through profilin, to G-actin to accelerate the polymerization process (Higgs, 2005; Goode and Eck, 2007; Breitsprecher et al., 2011; Courtemanche and Pollard, 2013; Courtemanche, 2018). Profilin regulates actin remodeling in cells by delivering G-actin to formin-bound actin filaments, while also inhibiting spontaneous actin polymerization by shielding the barbed ends of G-actin (Kovar et al., 2006). Interestingly, despite being modified, Mox-G-actin binds normally to profilin (Grintsevich et al., 2021). However, profilin-Mox-G-actin complexes do not fuel formin-mediated polymerization [Figure 2D (Grintsevich et al., 2021)]. Moreover, profilin alone inhibits the polymerization of Mox-actin, even when unoxidized actin seeds are present (Figure 2D (Grintsevich et al., 2021)). Thus, when combined with Mox-G-actin, profilin does not facilitate actin polymerization but inhibits it and further promotes F-actin disassembly (Grintsevich et al., 2021).

The Arp2/3 complex is a well-known nucleator and regulator of branched actin networks (Pollard, 2007; Pizarro-Cerda et al., 2017). The Arp2/3 complex consists of seven proteins, with Arp2 and Arp3/Arp3B being unconventional actin-related proteins that mimic a filamentous actin dimer (Pizarro-Cerda et al., 2017; Narvaez-Ortiz and Nolen, 2022). To attain an active actin dimer state, which acts as a nucleation site for polymerization of a new filament on the side of the mother filament, Arp2 and Arp3/Arp3B need to undergo conformation changes. These changes are mediated by cortactin and Wiskott Aldrich Syndrome protein (WASP) (Pollard, 2007; Pizarro-Cerda et al., 2017; Narvaez-Ortiz and Nolen, 2022). Notably, Arp3B is a target for MICAL-mediated oxidation, such that MICAL-2 oxidizes the Met293 of Arp3B (Galloni et al., 2021). This oxidation renders Arp3B inactive and promotes disassembly of branched actin networks (Galloni et al., 2021). The exact mechanism of debranching is unknown, but it could be due to an altered structure of the Arp2 and Arp3B complex, which no longer acts as a nucleation site for branch formation.

Overall, MICALs collaborate with other actin-binding proteins, changing actin polymerization/depolymerization dynamics and shifting the cell to impaired actin assembly conditions. Moreover, because of its reduced polymerization capacity, the MICAL-oxidized actin monomer is not reused in the actin assembly cycle. Thus, MICALs are potent actin disassemblers that combine with other proteins to dynamically tune their functional effectiveness.

## 6 Reversing the MICAL’s effects on F-actin: MsrB/SelR family reductases

In light of the identification of this new direct oxidation-dependent means to regulate actin cytoskeletal dynamics it was of great interest to determine whether these MICAL-mediated actin alterations were reversible. Notably, it was found that the MICAL’s actin modification (stereospecific oxidation of Met44 and Met47) was selectively counteracted by a family of stereospecific methionine sulfoxide reductase enzymes called MsrB/SelR (Hung et al., 2013; Lee et al., 2013). There are three MsrB/SelR enzymes in mammals (MsrB1, 2, and 3) and one in invertebrates (called SelR). In particular, MsrBs/SelRs can specifically reverse each of the MICALs effects on actin (Figure 2D), while other reductases, including other methionine sulfoxide reductase enzymes (MsrA) and chemical reducing agents such as DTT, cannot do that (Hung et al., 2013; Lee et al., 2013; Grintsevich et al., 2016; Wu et al., 2018). *In vitro* experiments with purified proteins reveal that MsrBs/SelRs restore Mox-actin polymerization and formin-assisted polymerization kinetics (Hung et al., 2013; Lee et al., 2013; Grintsevich et al., 2016; Wu et al., 2018; Bai et al., 2020; Grintsevich et al., 2021). MsrBs/SelRs also counteract MICALs *in vivo* effects, including on F-actin remodeling and actin-dependent cellular functions such as cell morphology, axon guidance, synaptogenesis, muscle organization, trafficking, and cytokinesis (Hung et al., 2013; Wu et al., 2018; Bai et al., 2020; Hamdan et al., 2020). Results of experiments support the point that while F-actin is a substrate for the MICALs, the MsrB/SelR’s reversing effect occurs on G-actin (Figure 2D (Hung et al., 2013; Lee et al., 2013)). Other points to consider are the relative rates of the MICAL’s oxidation reaction on actin *versus* the MsrB/SelR’s reduction reaction, and whether the catalytic activity of the MICAL’s *versus* the MsrB/SelR’s favors one reaction over the other. In short, experimental observations with purified proteins do not reveal major differences in the relative rates of the MICAL’s oxidation of actin *versus* the MsrB/SelR’s reduction of MICAL-oxidized actin. In other words, although the exact rates of the MICAL’s oxidation of actin have not been published, experimental studies (using antibodies to MICAL-oxidized actin and subtilisin digestion of F-actin after MICAL treatment) reveal that MICAL rapidly (essentially ∼ instantaneously) oxidizes filament subunits in a concentration-dependent manner (Hung et al., 2011; Hung et al., 2013; Grintsevich et al., 2016). MsrBs/SelRs quickly reverse this MICAL’s-oxidation of actin, and then actin polymerizes normally (Hung et al., 2013; Lee et al., 2013; Wu et al., 2018). *In vivo* observations are also consistent with these results with purified proteins. In particular, increasing MICAL levels induces dramatic effects on F-actin *in vivo* (Hung et al., 2010a; Wu et al., 2018), but the organization of F-actin in cells – with simultaneous increases in the MsrB/SelRs – indicates that the MsrB/SelRs serve to negate the MICAL’s effect (Hung et al., 2013; Wu et al., 2018). Lastly, although proteins (such as Plexins and Rabs) have been identified that prominently control/regulate the MICAL’s actions in cells (i.e., control the MICAL’s side of the reaction; see below for more details), little is known of what controls the MsrB/SelR side of the reaction in cells. Related to this, MICALs appear to exhibit a specific subcellular localization pattern (Hung et al., 2010a), while MsrB/SelRs are more broadly localized (Hung et al., 2013). This difference in localization may play a role in regulating MsrB/SelRs activity and effects on MICALs (including favoring one side of the reaction *versus* the other). The MICALs and MsrB/SelRs therefore comprise a specific reversible Redox system for robustly regulating actin dynamics.

## 7 Regulation of the MICAL’s activity

### 7.1 Activation of the MICALs: Relieving the MICAL’s autoinhibited state

MICAL Redox enzymes do not induce their effects on actin indirectly (e.g., *via* general effects on the cellular redox state or global H_2_O_2_ production), but locally target F-actin for disassembly (Hung et al., 2010a; Hung et al., 2013; Wu et al., 2018). So, an important question in this regard is how is the activity of the MICALs regulated in a localized manner. Work with purified proteins and *in vivo* reveals that MICALs are self-regulated through intramolecular interactions between their C-terminal ERM α-like domain and the N-terminal Redox and LIM domains [Figure 1B (Schmidt et al., 2008; Hung et al., 2010a; Giridharan et al., 2012; Vitali et al., 2016; Frémont et al., 2017)]. These intramolecular interactions mask the active site in the F-actin regulatory Redox domain of MICALs to render them catalytically inactive (Figure 1B).

**FIGURE 3:**
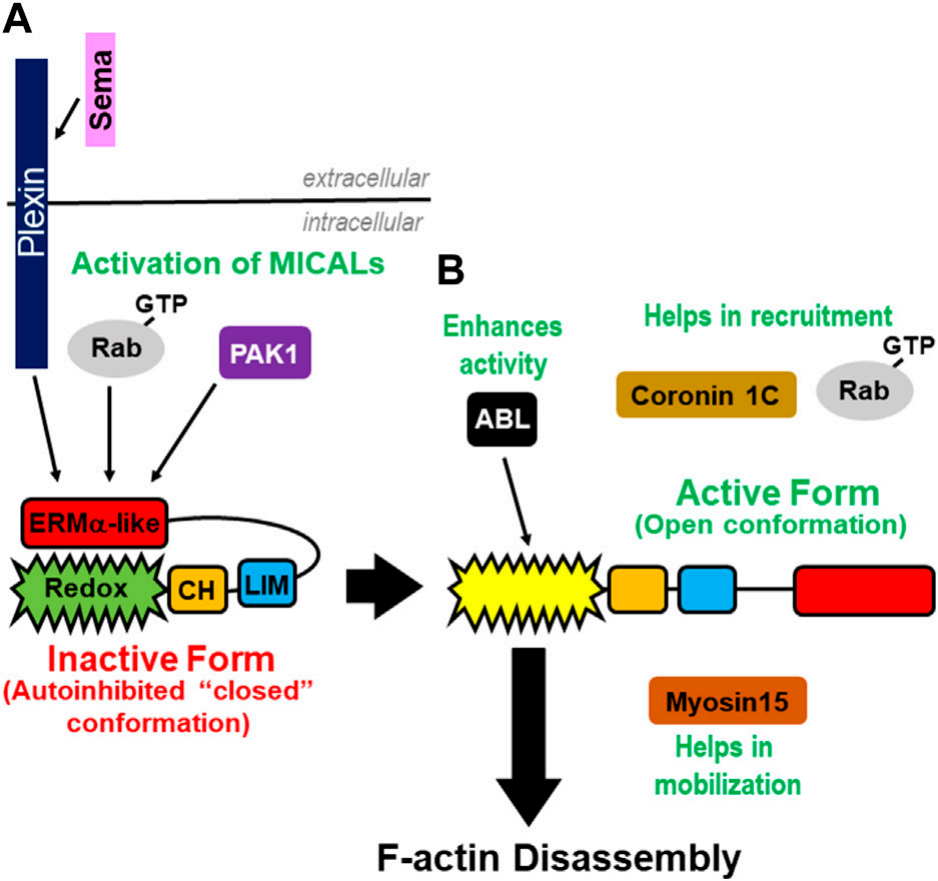
Regulation of the MICAL’s activity. **(A)** Plexins and GTP-bound Rab proteins bind the MICAL’s ERM α-like domain to relieve the MICAL’s autoinhibition. PAK1 kinases also play a role in relieving the MICAL’s autoinhibition by phosphorylating residues within the MICAL’s C-terminus. **(B)** Rabs and Coronin 1C are involved in the MICAL’s translocation and positioning. Myosin15 is involved in moving the MICAL’s into a new region to propagate its F-actin disassembly effects.

So how do MICALs become active? Physical interactions between MICALs and proteins in specific signaling pathways spatiotemporally relieve MICALs autoinhibition. In particular, the ERM α-like/PIR region of the MICALs directly interacts with the cytoplasmic region of Plexin transmembrane cell-surface receptors [Figure 3A (Terman et al., 2002)]. Plexins are receptors for one of the largest protein families of extracellular guidance cues, semaphorins (Semas), with over twenty family members conserved from invertebrates to humans (Alto and Terman, 2017). In the absence of Semas, Plexins’ cytoplasmic region is also autoinhibited, and results support a model that Sema binding to the extracellular portion of Plexin induces an allosteric change, which relieves this autoinhibition and activates Plexin (Pascoe et al., 2015). Activated Plexin then binds to the MICALs to relieve the MICAL’s autoinhibition and allow for the activation of its Redox domain to spatiotemporally regulate actin dynamics [Figures 3A,B (Terman et al., 2002; Schmidt et al., 2008)]. Further specificity, including precise regulatory mechanisms, is gained by different semaphorins and plexins utilizing different MICALs to exert their cellular effects, including on F-actin disassembly (Terman et al., 2002; Ayoob et al., 2006; Schmidt et al., 2008; Hung et al., 2010a; Morinaka et al., 2011; Aggarwal et al., 2015; Hou et al., 2015; Loria et al., 2015; Orr et al., 2017; Tominaga et al., 2019).

Specific Rab family small GTPases also bind and relieve the MICAL’s intramolecular autoinhibition (Figure 3A). In particular, multiple different Rab GTPases (including Rab1A/B, Rab7A, Rab8A/B, Rab10, Rab13, Rab15, Rab33B, Rab35, and Rab36) directly interact with different MICALs *via* MICALs’ ERM α-like/RBD region, and they do this primarily in their active GTP-bound state [Figure 3A (Weide et al., 2003; Fischer et al., 2005; Fukuda et al., 2008; Rai et al., 2016; Frémont et al., 2017; Liu et al., 2017; Tian et al., 2018; Gillingham et al., 2019; Hamdan et al., 2020; McGarry et al., 2022)]. Rab GTPases are well-known regulators of cellular functions, including as key vesicle trafficking proteins in endocytosis and exocytosis, and their direct interaction with MICALs releases the MICAL’s autoinhibition (Frémont et al., 2017; Esposito et al., 2019). Further specificity is gained by different MICALs interacting and utilizing different Rabs to recruit and activate them at specific locations to exert their cellular effects, including on F-actin disassembly [Figures 3A,B (Grigoriev et al., 2011; Deng et al., 2016; Frémont et al., 2017; Hamdan et al., 2020)]. Different Rab and MICAL-interacting proteins, including ELKS active-zone proteins (Grigoriev et al., 2011; Liu et al., 2017), NINL (Bachmann-Gagescu et al., 2015), MKLP1/Kif23 centralspindlin components/kinesin family proteins (Maliga et al., 2013; Liu et al., 2016; Liu et al., 2017), GRAF membrane tubulating proteins (Lucken-Ardjomande Hasler et al., 2020), TBC1D1 Rab GTPase activating proteins (GAPs) (Hook et al., 2020), alpha2-spectrin (Liu et al., 2016; Hamdan et al., 2020), and the MuSK and PAK1 serine/threonine kinases (Budayeva et al., 2022; McGarry et al., 2022) are also linked to Rab and MICAL interactions/cellular effects.

Thus, different modes of regulation help in the precise spatiotemporal activation of the MICALs, which is a prerequisite for normal cellular forms and functions [e.g., (Terman et al., 2002; Schmidt et al., 2008; Hung et al., 2010a; Frémont et al., 2017)]. Further, the dysregulation of the MICALs has a detrimental effect on cellular forms and functions. For example, constitutively-active forms of MICAL (e.g., those lacking the ERM α-like domain that serves an autoinhibitory function) induce widespread and excessive F-actin disassembly and marked abnormalities in cellular morphology/structure (Hung et al., 2010a; Giridharan et al., 2012; Wu et al., 2018) and disease (Dazzo et al., 2018). Related to this, without a C-terminal ERM α-like domain, MICAL-2 would not be autoinhibited in cells (i.e., it would be constitutively active in cells). Thus, further study is needed to determine if the MICAL-CL/Ebitein region/ERM α-like domain that is a part of the *MICAL-2* locus, is involved in MICAL-2’s autoinhibition (Terman et al., 2002; Hung and Terman, 2011).

### 7.2 Additional positive effectors of the MICALs

The MICAL’s effects have also been found to be enhanced through the action of specific signaling proteins. For example, vascular endothial-derived growth factor (VEGF), platelet-derived growth factor (PDGF), epidermal growth factor (EGF), and fibroblast growth factor (FGF) and their receptors have been linked to increasing the MICAL’s F-actin disassembly effects (Hou et al., 2015; Deng et al., 2016; Evans et al., 2017; Yoon et al., 2017; Barravecchia et al., 2019; McGarry et al., 2022). Further results reveal that they enhance the MICAL’s F-actin disassembly effects by working with Abl/Arg non-receptor tyrosine kinases, which phosphorylate specific tyrosine (Y) residues within the Redox domain of the MICALs (Y500 in Mical and Y445, Y463 in MICAL-2) [Figure 1A (Yoon et al., 2017; Yoon and Terman, 2018a; Shi et al., 2020; Zhang et al., 2022)]. Furthermore, at least in the case of Mical, this Abl phosphorylation increases its NADPH consumption activity in the presence of F-actin and potentiates its F-actin disassembly/repulsive activity [Figure 3B (Yoon et al., 2017)]. PAK1 kinase also works downstream of growth factors, and its activated form binds to the Redox and CH regions of MICAL-1 and phosphorylates MICAL-1 at specific serine (S) residues (S817, S960) within the PxxP and ERM α-like domain, respectively [Figure 1A (McGarry et al., 2022)]. This phosphorylation relieves MICAL-1’s autoinhibition and also increases Rab7A and Rab10’s interaction with MICAL-1 [Figure 3A (McGarry et al., 2022)]. These results (McGarry et al., 2022) also add to the work of others (Wang et al., 2018; Zhao et al., 2019; Wang et al., 2021a; Wang et al., 2021b; Qi et al., 2021) and link Rho family GTPases to the regulation of the MICAL’s effects. MICALs have also been associated with nerve growth factor (NGF) signaling, such that it induces MICAL-2’s F-actin disassembly activity in the nucleus to promote gene transcription through the serum response factor (SRF)/MRTF-A (Lundquist et al., 2014). MICALs have also been linked to regulating the effects of other extracellular ligands/receptors, including those of TGFβ/TGFR and Ephrins/Eph, on cell migration and F-actin disassembly (Liu et al., 2019; Shi et al., 2020; Jiang et al., 2021; Pu et al., 2021).

MICALs’ localization and action in specific subcellular regions is important for their F-actin/cellular effects. Towards this end, MICALs were recently found to interact with specific myosins, which are well-known regulators of cellular behaviors. Myosins regulate cell functions in two ways: they move/hold F-actin (i.e., myosins act as force generators/mechanical tethers) and/or they move processively along F-actin (i.e., myosins act as intracellular transports) (Coluccio and Biol, 2020). A specific myosin, Myosin 15 (Myo15), physically associates, transports, and broadens MICAL’s distribution to expand and directionally orient the MICAL’s F-actin effects [Figure 3B (Rich et al., 2021)]*.* MICALs also interact with members of another class of myosins, MyoVa (and MyoVb) (Niu et al., 2020). Yet, interestingly, in contrast to Myo15, which expands the MICAL’s distribution/F-actin disassembly, MICAL-1 was found not to be transported by MyoVa, but to be tethered to a specific spot and derail MyoVa and its cargo as MyoVa passed through that specific site (Niu et al., 2020). Thus, myosins are involved in at least two functions as it relates to the MICALs: 1) myosins expand MICALs-mediated F-actin disassembly and cellular remodeling and 2) myosins restrict MICALs-mediated F-actin disassembly and cargo unloading. Specific myosins may also interact with specific MICALs [e.g., MyoV interacts with MICAL-1 but not with MICAL-2 and 3 (Niu et al., 2020)] and control other aspects of MICALs’ functions [e.g., another myosin, Myo9, binds and promotes the nuclear export of MICAL-2 (Zhou et al., 2020)]. Other intracellular proteins have also been found to bind and recruit MICALs to specific subcellular regions. Coronin 1C, an actin regulatory protein, facilitates the recruitment of MICAL-2 to vaccinia-induced actin tails [Figure 3B (Galloni et al., 2021)]. This type of recruitment may be specific to different MICALs since coronin 1C recruits MICAL-2 but not MICAL-1 (Galloni et al., 2021).

MICALs have been found to interact also with other proteins, including most notably CasL/Nedd9 (Suzuki et al., 2002; Evans et al., 2017; Grauzam et al., 2018; Zhao et al., 2019) and the intermediate filament protein vimentin (Suzuki et al., 2002; Grauzam et al., 2018; Semelakova et al., 2019), but the specific roles of these interactions are unclear. Cortactin’s presence on actin branches is required for the enhanced MICAL-2’s effects on the dissociation of Arp3B-containing Arp2/3 complexes and the increased rate of actin network disassembly *via* yet unknown mechanisms (Galloni et al., 2021). MICALs have also been functionally linked to other proteins and signaling pathways, including ERK, PP2A, and RanBPM, among others, although the roles of these proteins/signaling pathways in regulating MICALs is unclear [e.g., (Togashi et al., 2006; Deng et al., 2018; Tao et al., 2019; Wolterhoff et al., 2020)]. Moreover, *in vitro* studies have suggested that MICALs’ activity depends on ionic strength and pH (Zucchini et al., 2011; Grintsevich et al., 2016), including that MICAL-1 shows higher catalytic turnover with increasing pH (Zucchini et al., 2011). Such changes may regulate MICALs’ effects *in vivo* – particularly given that intracellular alkaline pH promotes tumor progression and growth (White et al., 2017), and H+ transporters NBCn1 and NHE1 (and thereby intracellular alkaline pH) are upregulated in specific cancers [e.g., (Flinck et al., 2018)], and increased MICAL activity is associated with specific cancers (Yoon and Terman, 2018b) (see below).

### 7.3 Dampening the MICAL’s effects

Factors that dampen MICALs’ functions are also likely to play prominent roles in regulating the MICAL’s effects. Yet, besides MsrB/SelR, as described above, so far these negative regulators have been poorly defined. For example, an antagonistic relationship between MICAL-1 and NDR Kinase apoptotic signaling has been identified, but NDR kinases do not negatively regulate MICAL-1’s effects (Zhou et al., 2011). Notably, micro RNAs have been found that specifically target MICALs, including to promote actin polymerization (Tao et al., 2019; Torrini et al., 2019; Han et al., 2022). Future work is likely to reveal more effectors that work in opposition to MICALs.

## 8 The MICAL’s physiological and pathological functions

The MICAL’s role in robustly altering actin dynamics is critical for regulating specific cellular and tissue behaviors in the cytosol (often in close proximity to the plasma membrane) and in the nucleus. Furthermore, as described below, it is important to note that while the MICAL’s role *in vivo* was first identified in the nervous system (Terman et al., 2002), MICALs are broadly expressed and regulate the form, function, and dysfunction of multiple different types of cells and tissues. Yet, it is also clear that much remains to be learned about how broadly the MICALs are utilized *in vivo*, including a full understanding of their use by nature in different tissue systems, cell types, and specific cellular events. Below we provide a general overview of what is known of the MICAL’s *in vivo* functions. For additional coverage including detailed specifics of the MICAL’s *in vivo* functions, we refer readers to (Hung and Terman, 2011; Wilson et al., 2016; Manta and Gladyshev, 2017; Vanoni, 2017; Alto and Terman, 2018; Ortegón Salas et al., 2020; Haikazian and Olson, 2022; Rouyère et al., 2022).

### 8.1 Physiology: Cell biology, neurobiology, musculoskeletal biology, cardiovascular biology, and more

**FIGURE 4:**
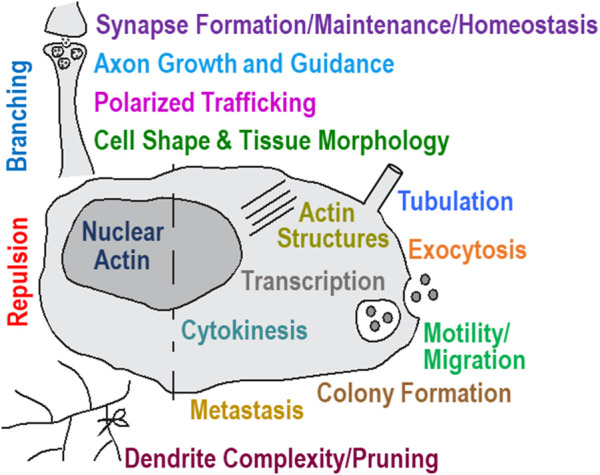
Cellular behavior controlled by the MICAL’s activity contributes to multiple functions and dysfunctions in numerous tissues. Modified from (Alto and Terman, 2018).

Cells undergo rapid reorganization during division, polarity, motility, navigation, and other behaviors by robustly inducing actin remodeling (Pollard and Borisy, 2003; Blanchoin et al., 2014). MICALs and their effects on F-actin structures have now emerged as robust modulators of these behaviors – being required and sufficient to alter the form and function of multiple different cells and tissues (Figure 4). In particular, MICALs were first identified for their functions in the nervous system – being required for the guidance of neuronal axons *in vivo* (Terman et al., 2002). Subsequent work has added to these guidance roles, as well as defined roles for the MICALs in neuronal growth cone morphology and axon extension (Beuchle et al., 2007; Schmidt et al., 2008; Hung et al., 2010a; Morinaka et al., 2011; Hashimoto et al., 2012; Hung et al., 2013; Lundquist et al., 2014; Van Battum et al., 2014). MICALs also regulate the morphology/complexity of neuronal dendrites (Kirilly et al., 2009; Loncle and Williams, 2012; Rumpf et al., 2014; Rode et al., 2018) and are important for establishing and regulating the connection between neurons (i.e., synaptic formation, organization, and activity) (Beuchle et al., 2007; Hung et al., 2013; Van Battum et al., 2014; Orr et al., 2017; Schaukowitch et al., 2017). MICALs regulate the migration of neurons (Bron et al., 2007), the trafficking of proteins into axons (Hamdan et al., 2020), the docking and fusing of vesicles at the plasma membrane (Grigoriev et al., 2011; Van Battum et al., 2014; Bachmann-Gagescu et al., 2015), and the morphology of neuro-mechanosensory system cells (e.g., *Drosophila* bristle cells, which are akin to the inner ear hair cells required for hearing in mammals) (Hung et al., 2010a; Hung et al., 2013).

MICALs also play important non-neuronal functions. They have been associated with regulating smooth, skeletal, and cardiac muscle organization, including being required to control F-actin organization in skeletal muscle (Beuchle et al., 2007; Schnorrer et al., 2010; Hung et al., 2013; Tao et al., 2019; Giarratana et al., 2020; Konstantinidis et al., 2020). They regulate the cardiovasculature, including in angiogenesis, vessel integrity, heart development/function, and lymphatic remodeling (Lundquist et al., 2014; Hou et al., 2015; Williams et al., 2017; Barravecchia et al., 2019; Konstantinidis et al., 2020; Erdmann et al., 2021; Francis et al., 2022). MICALs have been linked to kidney function (Aggarwal et al., 2015) and immune response (Lee et al., 2013). MICALs play important roles in other cell biological events in different cells, including specifying cell morphology [e.g., (Hung et al., 2010a; Hung et al., 2011; Giridharan et al., 2012; Hung et al., 2013; Hou et al., 2015; Wu et al., 2018)], migration [e.g., (Liu et al., 2019; Jiang et al., 2021; Pu et al., 2021)], proliferation [e.g., (Tao et al., 2019; Pu et al., 2021)], wound healing (Williams et al., 2017), membrane tubulation (Lucken-Ardjomande Hasler et al., 2020; Wang et al., 2021b), endocytosis/exocytosis/vesicle trafficking (Grigoriev et al., 2011; Bachmann-Gagescu et al., 2015; Liu et al., 2016), and cytokinesis, where they are required to control the F-actin cytoskeleton at abscission sites (Liu et al., 2016; Frémont et al., 2017; Bai et al., 2020). MICALs have also been defined for effects on cell-cell repulsion mediated by one of the largest families of cellular guidance cues, Semaphorins and their Plexin receptors (Terman et al., 2002; Schmidt et al., 2008; Hung et al., 2010a; Morinaka et al., 2011; Aggarwal et al., 2015; Hou et al., 2015; Yoo et al., 2016; Tominaga et al., 2019).

### 8.2 Pathology: Cancer, brain disorders, cardiovascular defects, and more

MICALs and their ability to robustly control the cytoskeleton have become increasingly linked to different pathologies, including that altered expression levels and SNPs/missense mutations for MICALs have been associated with numerous cellular dysfunctions (Figure 4) and disease. Below, we highlight a few of these studies in which functional analysis has been carried out. In particular, MICALs have been functionally linked to different types of cancers/cancer cells, including bladder (Ho et al., 2012), blood/leukemia (Corces-Zimmerman et al., 2014), brain/glioma (Pu et al., 2021), breast (Deng et al., 2016; Mariotti et al., 2016; Wang et al., 2017; Yoon et al., 2017; Deng et al., 2018; Tominaga et al., 2019; McGarry et al., 2021), colorectal (Gu et al., 2022), gastric (Mariotti et al., 2016; Zhao et al., 2019; Wang et al., 2021a; Qi et al., 2021), lung-related (Mariotti et al., 2016; Liu et al., 2019; Zhou et al., 2020; Wang et al., 2021b), pancreatic (Cai et al., 2022), prostate (Ashida et al., 2006), renal (Mariotti et al., 2016), and skin/mucous membrane (Loria et al., 2015; Grauzam et al., 2018; Zhang et al., 2022). Notably, the MICAL’s involvement in at least some of these cancers/cancer cells has been linked to its actions on F-actin. The MICALs have also been prominently linked to neurological and mental health disorders, including neurodegeneration (Prifti et al., 2022), spinal cord injury (Pasterkamp et al., 2006; Xu et al., 2021), and epilepsy (Dazzo et al., 2018). Most notably, heterozygous missense mutations in the Redox domain (Gly150Ser) and C-terminal ERM α-like domain (Ala1065fs, deletion of last three amino acids, and addition of 59 extra residues) generate changes in MICAL-1 that have been linked to Autosomal-Dominant Lateral Temporal Epilepsy (Dazzo et al., 2018). In particular, it is thought that these dominant mutants generate constitutively active MICAL-1 and its effects on cells are consistent with increased F-actin cytoskeletal disassembly (Dazzo et al., 2018). Since MICAL-1 is also expressed in other tissues besides the brain, it is interesting to consider if these heterozygous missense mutations lead to comorbid pathologies in other tissues/behaviors, or whether the brain may be particularly susceptible to having these heterozygous missense *MICAL-1* alleles (i.e., only one normal copy of *MICAL-1* present). MICALs have been functionally linked to cardiovascular abnormalities, including heart arrhythmias, pathologic responses to cardiac stress, myocardial hypertrophy, endothelial and blood-brain barrier (BBB) permeability, and contributing to effects seen with mutations in cardiac actin (Hou et al., 2015; Konstantinidis et al., 2020; Erdmann et al., 2021; Zhao et al., 2021). Notably, the MICAL’s involvement in at least some of these cardiovascular defects has been linked to its actions on F-actin. MICALs have also been functionally linked to renal disease/diabetic nephropathy through effects on the F-actin cytoskeleton (Aggarwal et al., 2015). Less well-defined functional links between MICALs and muscular dystrophy (Marotta et al., 2009), fibrosis (Zhao et al., 2020; Jiang et al., 2021), autoimmune disorders (Johar et al., 2015), skin aging (Han et al., 2022), and viral infection (Shapira et al., 2009; Mitchell et al., 2013) have been observed. Lastly, as described above, the sites that the MICALs modify on actin (Met44 and/or Met47) have been linked to different diseases, including nemaline myopathy, CAP myopathy, aortic aneurisms, hypertrophic cardiomyopathy, intestinal hypoperistalsis, Baraitser–Winter cerebrofrontofacial syndrome, and ductus arteriosus (Laing et al., 2009; Hung et al., 2010b; Hoffjan et al., 2011; Zou et al., 2013; Regalado et al., 2014; Wangler et al., 2014; Yates et al., 2017; Zhang et al., 2019). It is also worth considering that some of the defects associated with mutations in the family of enzymes that reverse the MICAL’s effects, MsrBs/SelR’s, may result from increased effects of the MICALs on F-actin [see (Tarrago et al., 2022) for coverage of those defects].

## 9 Summary and conclusion

The MICAL family of proteins was discovered a little over 20 years ago, and with the uncovering of their enzymatic domain and activity, specific targeting of F-actin as a substrate, and their role in numerous cellular and tissues systems, they have now matured from proteins of unknown function to ones of significant interest in biomedical research that are being pursued by multiple laboratories. Yet, much remains to be learned about the MICALs and the reversible Redox-driven mechanism they use to regulate F-actin dynamics. We need to further define MICALs’ interactions with F-actin and 1) their effects on different F-actin networks, 2) their roles in different cells, tissues, and biological events, 3) their ability to interact with and regulate the effects of classical actin regulatory and signaling proteins, and 4) their interactions with proteins that may dampen their effects. Numerous studies have revealed altered expression levels and SNPs/missense mutations for MICALs in diverse diseases, but our understanding of these changes (including if, how, and the molecular/cellular basis for the MICAL’s possible involvement in these pathologies) is still poor and in need of further exploration. Thus, future studies should also focus on these areas of critical biomedical importance. MICALs have now emerged as a phylogenetically-conserved family of proteins with surprising functions, unexpected mechanisms of action, and crucial *in vivo* importance. The continued study of these biomedically significant proteins is likely to yield further surprising and unexpected discoveries.
